# Androgen potentiates the expression of FSH receptor and supports preantral follicle development in mice

**DOI:** 10.1186/s13048-019-0505-5

**Published:** 2019-04-04

**Authors:** Yuya Fujibe, Tsuyoshi Baba, Sachiko Nagao, Sayaka Adachi, Keiko Ikeda, Miyuki Morishita, Yoshika Kuno, Masahiro Suzuki, Masahito Mizuuchi, Hiroyuki Honnma, Toshiaki Endo, Tsuyoshi Saito

**Affiliations:** 1 Present Address: Department of Obstetrics & Gynecology, Sapporo Medical University, South 1 West 16, Sapporo, Hokkaido 060-8543 Japan; 2 Sapporo ART clinic, North 7 West 4, Sapporo, Hokkaido 060-0807 Japan

**Keywords:** Androgens, Follicle-stimulating hormone, Follicular development, Gene expression, Rodents

## Abstract

Hyperandrogenism is one of the cardinal symptoms in polycystic ovary syndrome and plays a key role in the pathogenesis of polycystic ovary syndrome. However, the precise effects and mechanisms of excess androgen during follicular development are still unclear. Here we investigated the effects of androgen on mouse follicle development in vitro. Androgen did not affect the growth of follicles smaller than 160–180 μm in the presence of follicle-stimulating hormone (FSH). However, in the presence of low FSH, androgen supported the growth of follicles larger than 160–180 μm, a size at which growing follicles acquire FSH-dependency. Androgen did not change the mRNA expression of various growth-promoting factors but did increase mRNA expression of the FSH receptor. We suggest that androgen has a positive impact on follicle development by augmentation of the actions of FSH. Therefore, FSH-responsive but FSH-independent follicles grow in the presence of a certain level of FSH or androgen, and androgen compensates for FSH deficiency in FSH-dependent follicles.

## Background

Polycystic ovary syndrome (PCOS) is a common endocrine disorder in women of reproductive age, with a reported prevalence of 8–20% [1]. The features of PCOS are chronic anovulation due to arrested follicle development, hyperandrogenism (clinical/biochemical) and polycystic ovary morphology (PCOM). Several criteria have been suggested for diagnosing PCOS. The National Institute of Health criteria proposed in 1992 include only two requirements: the presence of clinical and/or biochemical hyperandrogenism, and chronic anovulation [2]. In 2003, the European Society of Human Reproduction and Embryology/American Society for Reproductive Medicine developed a larger set of criteria for PCOS that require two of three features: anovulation or oligoovulation, clinical and/or biochemical hyperandrogenism and PCOM on ultrasound. The Androgen Excess and PCOS Society stated excess androgen as a requirement for PCOS diagnosis, accompanied by oligomenorrhea, PCOM or both [3, 4]. Thus, all major classifications have indicated a central role for excess androgen in the pathogenesis of PCOS.

Several animal models have been developed to examine the effects of excessive androgen on follicle development. However, the pathological changes in rodent models in response to androgen administration are different from the ovarian features observed in human PCOS. For example, several studies showed that in vivo exposure of androgen in rodents causes follicle atresia and ovarian atrophy [5–7]. Thus, experimental animal models exposed to androgens do not completely mimic PCOS in humans.

Follicles consist of oocytes, granulosa cells and theca cells. Follicle development and steroidogenesis are influenced by the interactions of theca cells and local growth factors. In addition, gonadotropins are key hormones that regulate follicular development. To examine the effects of androgen on follicle development without the influence of hypothalamic and pituitary functions, several studies have used in vitro culture of single follicles. Follicle development begins with the activation of primordial follicles, which develop into primary follicles, followed by development into secondary follicles, preantral follicles and eventually antral follicles [8]. The transition from secondary follicles to antral follicles is significant in the acquirement by antral follicles of the dependence on follicle stimulating hormone (FSH) for follicle development. In PCOS, there is an increased density of small pre-antral follicles and an increased proportion of early growing follicles [9]. To better clarify the effect of androgen on follicle development, secondary follicles might be a useful tool.

In this study, we investigated the direct effects of androgen on secondary follicles without interaction from the pituitary and hypothalamus. We examined the effects of androgen in low FSH and high FSH conditions on mouse secondary follicle development, steroidogenesis and expression levels of local growth factors.

## Methods

### Animals

Female ICR mice were obtained from Sankyo Labo Service Corporation (Sapporo, Japan) and housed under specific pathogen free conditions. ICR mice have high fertility and are easy to be handled for their size and meekness. They are commercially available as a closed colony animal and are used for various studies including in vitro culture of ovarian follicles. Therefore we chose ICR mice. Mice were handled according to the guidelines provided by Sapporo Medical University and the Scientists Center for Animal Welfare. All experimental protocols were approved by the Institutional Animal Care and Use Committee.

### Follicle isolation and culture

Six-week-old female ICR mice (*n* = 7) were killed by intraperitoneal injection of pentobarbital (120 mg/kg). Secondary follicles (100–160 μm in diameter) were mechanically isolated using 30-gauge needles. Follicles with an intact basement membrane, clear granulosa cell layers and oocyte and centrally located round oocytes were selected for analysis. Each follicle was individually placed into wells of a 48-well multiple cell-repellent surface plate (Greiner Bio-One, Kremsmunster, Austria) containing 500 μl of alpha minimum essential medium (Thermo Fisher Scientific, MA, USA) supplemented with 5% fetal bovine serum (Australia Source; CORNING, NY, USA), 10 μg/ml insulin, 5.5 μg/ml transferrin, 6.7 ng/ml sodium selenite and 200 IU/ml penicillin (Thermo Fisher Scientific). Follicles were cultured at 37 °C in a humidified environment with 5% CO_2_. Every other day, half of the culture medium was exchanged with fresh medium and stored at − 20 °C for hormone measurement. Culture was continued for 13 days.

### Secondary follicle culture conditions

Secondary follicles were cultured under low FSH or high FSH concentrations for experimental analyses. Low FSH was defined as 33 mIU/ml, as a previous study showed that the minimal FSH concentration required to elicit a maximal FSH-induced growth response was 67 mIU/ml [10]. Secondary follicles (12 follicles/mouse/group) from four mice were randomly assigned to four groups: 1) CTRL group: base media plus low FSH (33 mIU/ml FSH) and DHT vehicle (100% ethanol); 2) DHT 50 group: CTRL media plus DHT 50 ng/ml; 3) DHT 500 group: CTRL media plus DHT 500 ng/ml; and 4) DHT 1250 group: CTRL media plus DHT 1250 ng/ml. Culture media was supplemented with FSH from Sigma-Aldrich (MO, USA) and DHT from Tokyo Kaken (Tokyo, Japan).

High FSH condition was defined as 100 mIU/ml. Secondary follicles (12 follicles/mouse/group) from three other mice were randomly assigned to four groups: 1) CTRL group: base media plus high FSH (100 mIU/ml FSH) and DHT vehicle (100% ethanol); 2) DHT 50 group: CTRL media plus DHT 50 ng/ml; 3) DHT 500 group: CTRL media plus DHT 500 ng/ml; and 4) DHT 1250 group: CTRL media plus DHT 1250 ng/ml.

### Follicle survival and growth

Follicle survival and growth were assessed at day 1, 6 and 13 using an SMZ18 inverted microscope system (Nikon, Tokyo, Japan). Follicles were considered to be degenerating if the oocyte became dark or ejected outside of the follicle, if granulosa cells were dark and lysed or if the diameter of the follicle decreased. The diameter of each follicle was determined by averaging two measurements, perpendicular to each other, using NIS Elements Documentation D 3.22.00 (Nikon).

### Measurement of estradiol (E2) and progesterone (P4)

Concentrations of E2 and P4 in culture media were measured at day 13 after culture in low or high FSH condition. E2 levels were measured by an estradiol ELISA test kit (Neogen, MI, USA), with a detective range of 0–2.0 ng/ml, according to the manufacturer’s instructions. P4 levels were measured by a progesterone kit (Neogen), with a detective range of 0–20 ng/ml, according to the manufacturer’s instructions.

### RNA extraction, reverse transcription and real time quantitative polymerase chain reaction (qPCR)

At day 3 of culture, four to six secondary follicles in each experimental group were analyzed for mRNA expression. Follicles were ruptured by 30-gauge needles, and follicle wall and cumulus cells were collected for RNA extraction. Total RNA was isolated using the Absolutely RNA Nanoprep Kit (Agilent, CA, USA) according to the manufacturer’s instructions. Complementary DNA was synthesized using 1 μg of total RNA using Super Script II Reverse Transcriptase (Thermo Fisher Scientific). qPCR was carried out using the TaqMan gene expression assay and AB StepOne Plus Real-Time PCR System (Applied Biosystems, CA, USA). The gene expressions of FSH receptor (*Fshr*) (Assay ID: Mm00442819_m1), androgen receptor (*Ar*) (Assay ID: Mm00442688_m1), aromatase (*Cyp19a1*) (Assay ID: Mm00484049_m1), phosphatase and tensin homolog (*Pten*) (Assay ID: Mm00477208_m1), anti-Müllerian hormone (*Amh*) (Assay ID: Mm00431795_g1), AMH receptor 2 (*Amhr2*) (Assay ID: Mm00513847_m1), bone morphogenetic protein (*Bmp*) 2 (Assay ID: Mm0132882_m1), *Bmp6* (Assay ID: Mm 01332882_m1), *Bmp7* (Assay ID: Mm00477650_m1), activin A receptor type 1 (*Acvr1*) (Assay ID: Mm01331069_m1), *BMP* receptor type 1a (*Bmpr1a*) (Assay ID: Mm00477650_m1) and BMP receptor type 1b (*Bmpr1b*) (Assay ID: Mm03023971_m1) were analyzed by the ΔΔCt method. Glyceraldehyde 3-phosphate dehydrogenase (*Gapdh*) (Assay ID: Mm99999915_g1) was used for normalization. The amplification program included 40 cycles of denaturation at 95 °C for 15 s and 60 °C for 60 s. All reactions were run in triplicate.

### Statistical analysis

Data are presented as mean ± standard error of the mean. Statistical significance was determined using one-way analysis of variance (ANOVA) and Student-Newman-Keuls post hoc analysis with SigmaPlot version 13.0 (Systat Software, CA, USA) for data comparison among different treatment groups. Differences were considered significant at *P* < 0.05.

## Results

We evaluated the effects of androgen on early folliculogenesis by examining the effects of androgen under two sets of conditions, low and high FSH concentrations. Low FSH concentration was defined as 33 mIU/ml, as a previous study showed that the minimal FSH concentration required to elicit a maximal FSH-induced growth response was 67 mIU/ml [10]. We defined high FSH conditions as 100 mIU/ml.

### Androgen significantly reduced follicle survival rates but did not impact follicle growth under low FSH conditions

**Fig. 1:**
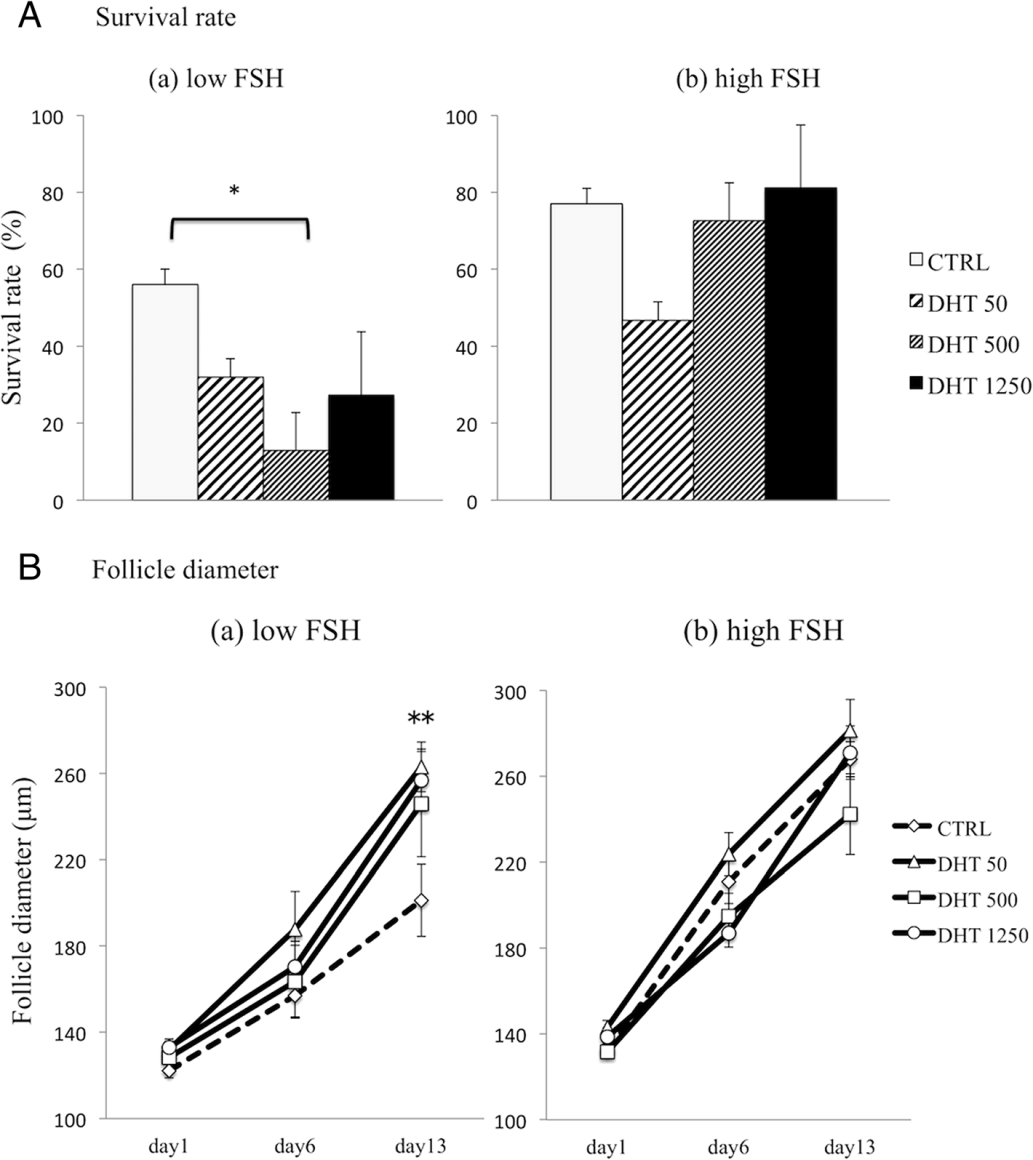
Follicle survival rates (**a**) and growth rates (**b**) under low or high FSH conditions. Follicles were cultured under (*a*) low FSH (33 mIU/ml) or (*b*) high FSH (100 mIU/ml) with DHT as indicated (50, 500, 1250 ng/ml). Controls (CTRL) were cultured in base media plus FSH and DHT vehicle (100% ethanol). Follicle survival was calculated at day 13 of culture (**a**), and follicle growth was monitored at the indicated time points (**b**). Data are expressed as mean ± standard error of the mean. Statistical analysis was performed using one-way ANOVA and Student-Newman-Keuls post hoc analysis for data comparison among different treatment groups. *P = 0.026, **P = 0.020

We first evaluated follicle survival rates in follicles treated with increasing concentrations of DHT (50 ng/ml, 500 ng/ml and 1250 ng/ml) under low FSH conditions (Fig. 1a(*a*)). The survival rates of follicles cultured with DHT 500 ng/ml was significantly lower than that in the CTRL group (12.9 ± 9.87% vs. 56.0 ± 3.95%, respectively; *P* = 0.026). Rates in the DHT 50 ng/ml and 1250 ng/ml groups (31.9 ± 4.83% and 27.3 ± 16.4%, respectively) tended to be lower than that in the CTRL group, but without significance (*P* = 0.078 and *P* = 0.096, respectively). There were no significant differences in follicular survival rates among the DHT groups.

We also examined the average diameters in surviving follicles cultured with DHT and low FSH at various time points. No significant differences were observed among the groups at days 1 and 6 (*P* = 0.155 and *P* = 0.374, respectively) (Fig. 1b(*a*)). However, at day 13, the average diameters of follicles under all DHT conditions (263.05 ± 11.51 μm in the DHT 50 ng/ml group, 245.79 ± 24.51 μm in the DHT 500 ng/ml group, and 256.82 ± 14.48 μm in the DHT 1250 ng/ml group) were significantly larger than that in the CTRL group (201.16 ± 16.65 μm; *P* = 0.020). The difference was not dependent on DHT concentration. These results indicate that androgen compensated for the lack of FSH and supported the growth of preantral follicles larger than 160–180 μm.

### Measurements of E2 and P4 under low FSH conditions

**Fig. 2:**
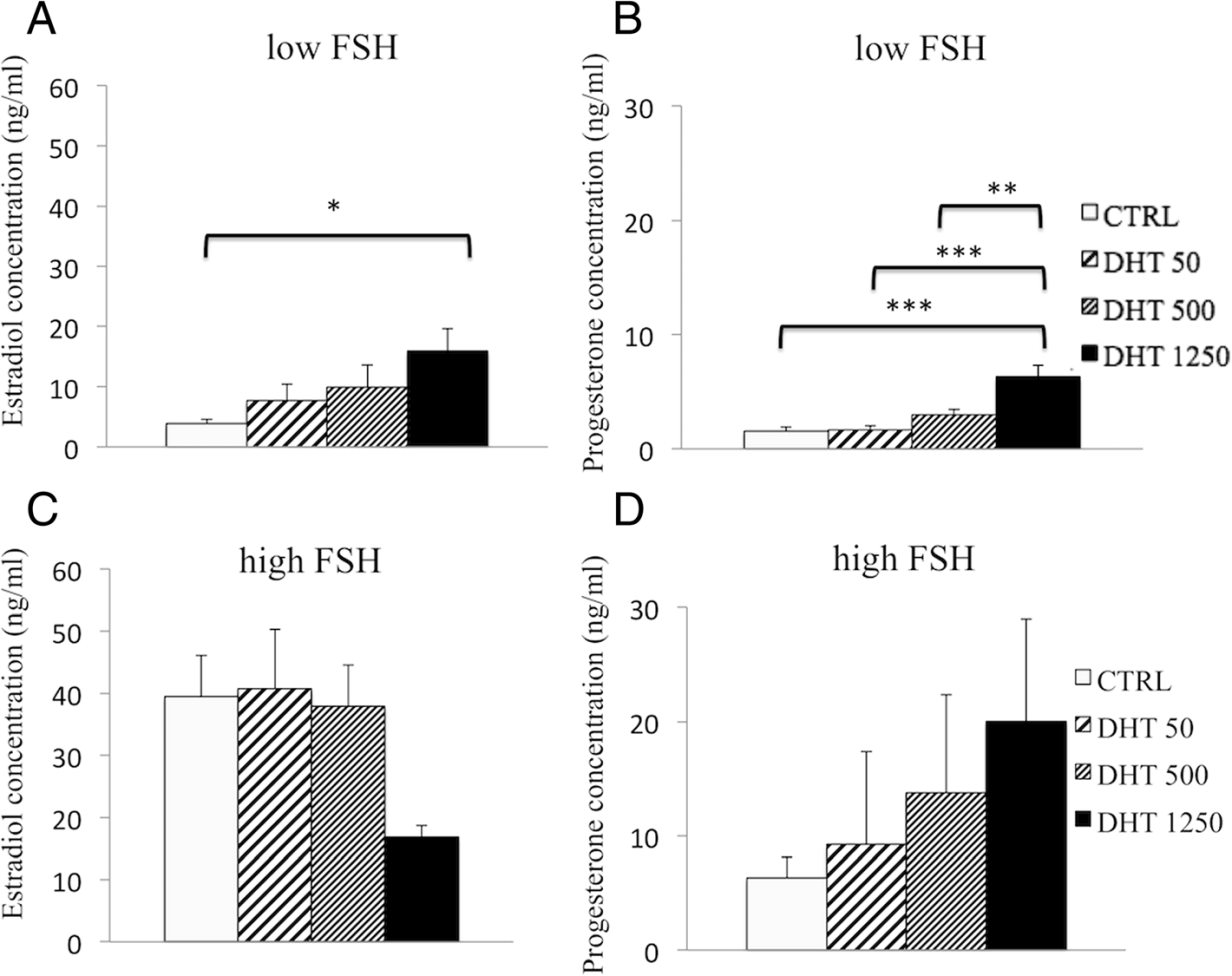
Estradiol (E2) and progesterone (P4) concentrations in culture media under low and high conditions. **a** E2 and **c** P4 concentrations in culture media under low FSH (33 mIU/ml); **b** E2 and **d** P4 concentrations in culture media under high FSH (100 mIU/ml). Follicles were cultured as indicated and described in Fig. 1. Data are expressed as mean ± standard error of the mean. Statistical analysis was performed using one-way ANOVA and Student-Newman-Keuls post hoc analysis for data comparison among different treatment groups. *P = 0.022, **P = 0.003, ***P < 0.001

We next examined the effect of DHT on E2 and P4 concentrations under low FSH conditions. Overall, we observed that increasing concentrations of DHT increased E2 production by preantral follicles (Fig. 2a), with a significant difference in E2 concentrations in the DHT 1250 group compared with the CTRL group (15.87 ± 3.77 ng/ml vs. 3.84 ± 0.78 ng/ml, respectively; *P* = 0.022).

Similarly, P4 levels also significantly increased in response to increasing DHT concentrations (Fig. 2b). We observed significant differences in P4 levels in the DHT 1250 group compared with the other three groups (CTRL vs. DHT 1250, *P* < 0.001; DHT 50 vs. DHT 1250, *P* < 0.001; DHT 500 vs. DHT 1250, *P* = 0.003).

### Transcriptional changes of various genes under low FSH conditions

**Fig. 3:**
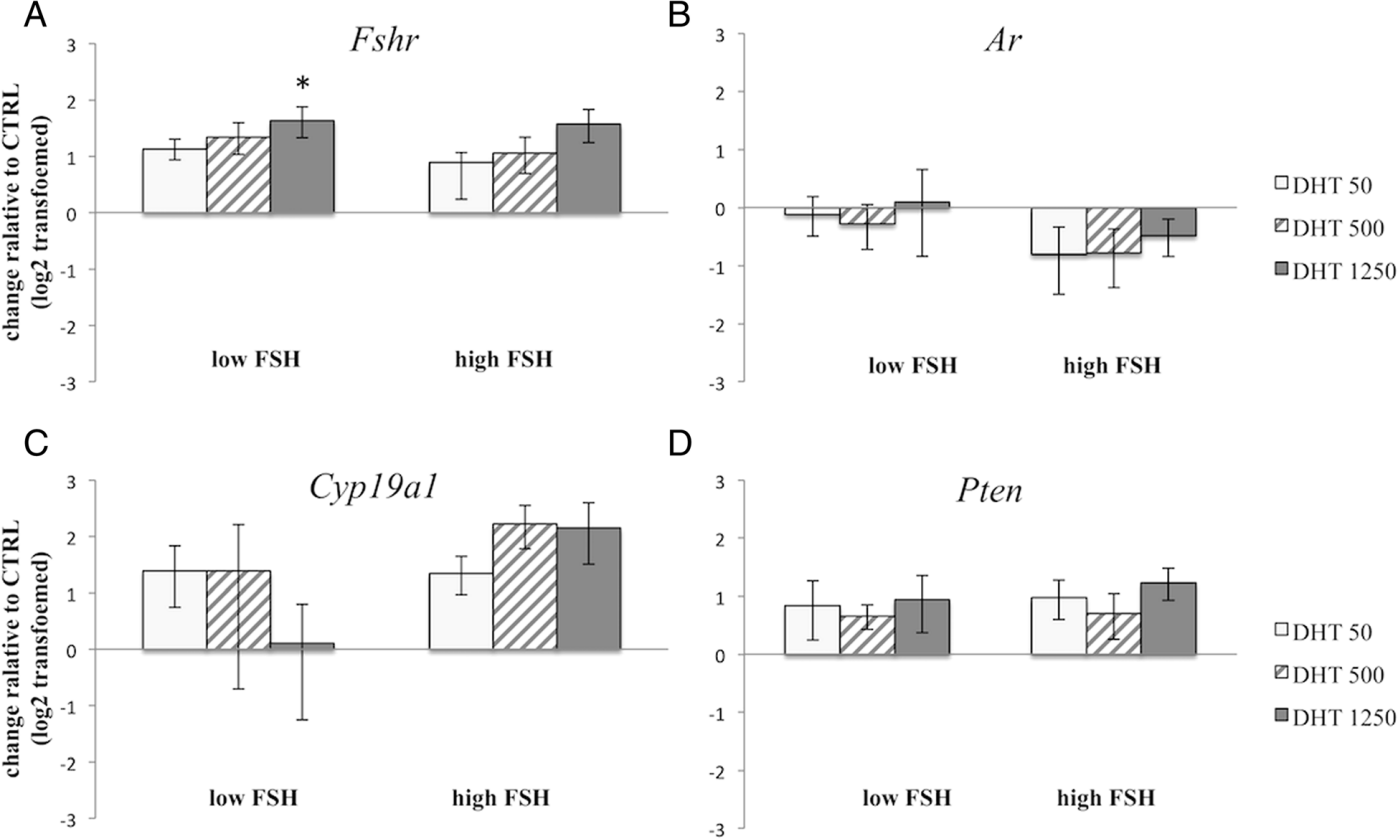
Effects of DHT on gene expressions of hormone receptors, *Cyp19a1* and *Pten* under low and high FSH conditions. Isolated follicles were cultured for 3 days and analyzed for gene expressions of (**a**) *Fshr*, (**b**) *Ar*, (**c**) *Cyp19a1* and (**d**) *Pten*. Follicles were cultured as indicated and described in Fig. 1. Data are expressed as mean ± standard error of the mean. Data have been log_2_ transformed and DHT groups were compared with the CTRL group. Statistical analysis was performed using one-way ANOVA and Student-Newman-Keuls post hoc analysis for data comparison among different treatment groups. *P = 0.026

We next examined changes in gene expressions in response to DHT under low FSH conditions. Exposure to DHT at 1250 ng/ml under low FSH conditions significantly increased *Fshr* mRNA expression (Fig. 3a) compared with controls, with the *Fshr* mRNA level in the DHT 1250 ng/ml group three-fold higher than that in the CTRL group (*P* = 0.026). However, no significant differences were detected in *Ar* mRNA, *Cyp19a1* mRNA or *Pten* mRNA levels among all groups (Fig. 3b–d).

**Fig. 4:**
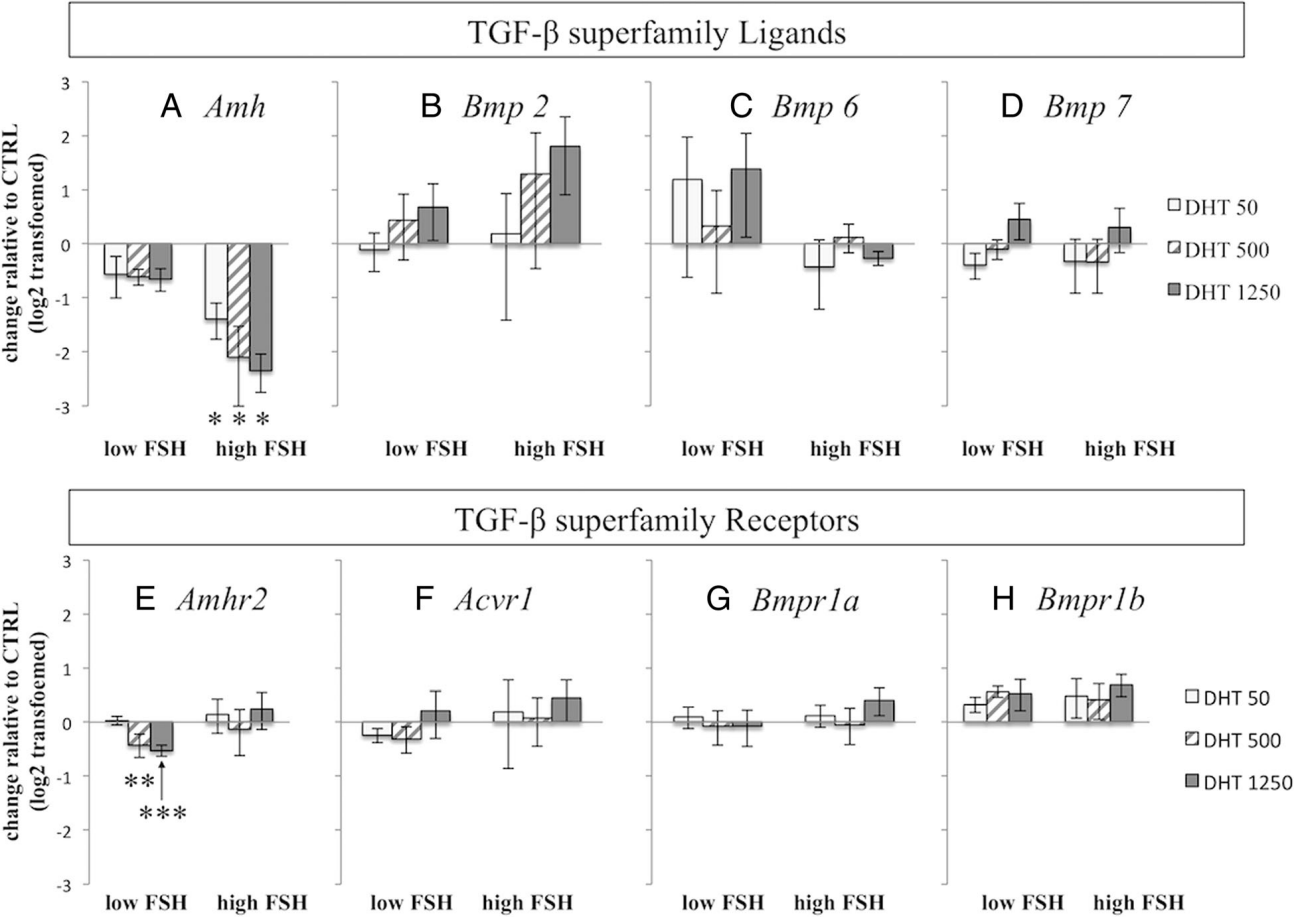
Effects of DHT on gene expressions of TGF-β superfamily ligands and receptors under low and high FSH conditions. Isolated follicles were cultured for 3 days and analyzed for mRNA expression levels of (**a**) *Amh*, (**b**) *Bmp2*, (**c**) *Bmp6*, (**d**) *Bmp7*, (**e**) *Amhr2*, (**f**) *Acvr1*, (**g**) *Bmpr1a* and (**h**) *Bmpr1b*. Follicles were cultured as indicated and described in Fig. 1. Data are expressed as mean ± standard error of the mean. Data have been log_2_ transformed and DHT groups were compared with the CTRL group. Statistical analysis was performed using one-way ANOVA and Student-Newman-Keuls post hoc analysis for data comparison among different treatment groups. *P < 0.001, **P = 0.027, ***P = 0.035

We also examined the mRNA levels of several TGF-β superfamily ligands and receptors (Fig. 4a–h). The expression levels of *Amhr2* mRNA in the DHT 500 ng/ml and 1250 ng/ml groups were significantly lower than that in the CTRL group (*P* = 0.027 and *P* = 0.028, respectively) (Fig. 4e). However, the relative expression levels were quite low and the application of these findings may be limited. We did not detect any other significant differences in the other genes among the groups.

### Androgen had no impact on follicle survival rates or growth under high FSH conditions

We also examined the impact of DHT on follicle survival rates and growth under high FSH conditions. We did not detect any statistically significant differences in follicle survival among the DHT groups compared with the CTRL group (Fig. 1a(*b*)). Furthermore, in contrast to the growth rates under low FSH conditions, follicles in all DHT groups cultured in high FSH conditions showed similar growth even through day 13 of culture (Fig. 1b(*b*)). The follicle diameters at day 13 were 267.9.32 ± 8.14 μm in the CTRL group, 281.37 ± 14.48 μm in the DHT 50 ng/ml group, 242.38 ± 18.86 μm in the DHT 500 ng/ml group, and 271.01 ± 12.47 μm in the DHT 1250 ng/ml group. These findings indicated that androgen did not affect preantral follicle development in the presence of abundant FSH. Interestingly, the average follicle diameters in all groups under both low and high FSH conditions at day 13 were over 240 μm, except in the CTRL group cultured under low FSH conditions.

### Measurements of E2 and P4 under high FSH conditions

We also examined the effect of various concentrations of DHT on E2 and P4 under high FSH conditions. However, we did not detect any statistically significant difference in culture media E2 concentrations among the groups (Fig. 2c). Similarly, no significant differences were detected in culture media P4 levels among the groups (Fig. 2d). Overall, the E2 and P4 levels under high FSH conditions appeared higher than those in low FSH conditions.

### Transcriptional changes of various genes under high FSH conditions

We examined the expressions of *Fshr*, *Ar*, *Cyp19a1* and *Pten* mRNA under high FSH conditions but did not detect any statistically significant differences in any gene among the groups (Fig. 3). In addition, the mRNA expressions of most ligands and receptors of the TGF-β superfamily did not show significant changes among the groups (Fig. 4a–h). However, DHT treatment significantly decreased *Amh* mRNA expression compared with levels in the CTRL group (*P* < 0.001, all DHT groups vs. CTRL) (Fig. 4a).

## Discussion

Our qPCR results indicate that DHT may exert pro-proliferative effects on mouse preantral follicles via increased *Fshr* mRNA expression, as demonstrated by Sen et al. [11]. In contrast, Laird et al. reported somewhat controversial data. The authors showed that DHT stimulated mouse secondary follicle growth in the presence of FSH and the effect of DHT was positively correlated with concentration of DHT [12]. However, DHT-treated follicles larger than 130 μm in diameter at the start of culture did not grow more in the presence of sufficient FSH. These findings indicate that androgen affects both secondary follicles (< 130 μm) and preantral follicles (> 160–180 μm), and these effects might occur via different pathways. In the present study, the proportions of follicles with a diameter larger than 130 μm were relatively high and therefore androgen action during the initial several days of culture (during secondary follicle stage) was limited.

Our present research demonstrates that androgen action on follicle growth is influenced by FSH concentration, and androgen affects mouse preantral follicles via increased FSH action. These results indicate that FSH-dependency in follicles is an essential factor for androgen to potentiate follicle development. In this context, our results also indicate that mouse follicles seem to acquire FSH-dependency once they reach a size of 160–180 μm (the preantral stage). A previous study on gonadotropin releasing hormone-deficient mice suggested that follicles could not grow beyond type 5b [13], as classified by Pedersen and Peters [14]. Type 5b follicles are preantral follicles with multi-layered granulosa cells and are usually 150–180 μm in diameter [15]. These reports indicate that follicles over 150–180 μm in diameter are required for follicle growth in response to gonadotropin. Our results are consistent with these studies. A similar transition of follicle sensitivity to FSH is observed in human, although human follicles acquire FSH-dependency after the early antral stage. In human, secondary follicles respond to FSH, however, follicles can develop to the antral stage in the absence of FSH, suggesting that the secondary follicle stage is not FSH-dependent but FSH-responsive [16, 17]. Furthermore, antral follicle growth is dependent on FSH, indicating that a high level of FSH promotes antral follicle growth. Similar phenomena were observed in non-human primates [18, 19]. Together these results suggest that the difference in follicle development between mice and humans is the timing for acquiring FSH-dependency.

In this study, we found that the follicle survival rates tended to be lower in the low FSH groups than those in the high FSH groups. This suggests that secondary follicles need sufficient FSH to survive and develop. Kreeger et al. showed that the survival rate of multilayered but not two-layered secondary follicles from mice was significantly lower in the absence of FSH [15]. Judging from present data on follicle growth, androgen seems to potentiate the action of FSH. In this context, follicles cultured with DHT supplementation are likely to show increased survival. However, our results did not show any positive effects of androgen on follicle survival. Laird et al. suggested that the combined treatment of FSH and DHT (but not individual treatment alone) disrupted the basal lamina surrounding the granulosa cell layer [12]. Kreeger et al. also showed that FSH concentrations greater than 25 mIU/ml reduced multilayered secondary follicle survival [15]. These results show that FSH does not always support follicle survival in a dose-dependent manner. This observation might be because theca cell layers are vulnerable to the drastic proliferation of granulosa cells within follicles caused by abundant FSH. Alternatively, increased metabolic activities in grown follicles may require more frequent replacement of culture media to survive.

Several hormones and local growth factors are known to affect follicle development. BMPs and AMH, members of the TGF-β superfamily, are produced by granulosa cells and theca cells and act as regulators of proliferation [10, 12]. The PTEN tumor suppressor protein regulates follicle development and steroid hormone production. *Pten* mutation in theca cells causes androgen excess and ovarian enlargement [20], and targeted disruption of *Pten* in granulosa cells leads to increased proliferation and survival [21]. AMH acts as a promoter of secondary follicle development [22]. Androgen affects various hormones and growth factors. However, the interactions among these factors are complex. Androgen affects some factors to promote follicle development and negatively impacts others to diminish folliculogenesis. To elucidate the effects of androgen on the follicular environment, we examined the mRNA expression levels of *Bmps*, *Amh*, their receptors and *Pten*. Our results also suggest that androgen suppresses *Amh* transcription; however, our experiment did not show any relationship between downregulation of *Amh* and secondary follicle development. It is because the addition of DHT significantly decreased *Amh* expression in the presence of high FSH, nevertheless, it did not alter secondary follicle growth. These results suggest that androgen action on preantral follicles is mainly due to increased FSHR mRNA expression. Endocrine, paracrine, and autocrine factors secreted by follicles and surrounding environment are believed to exert follicle development. Therefore in vivo environment might be required for assessing transcriptional changes in the genes.

## Conclusions

Mice preantral follicles larger than 160–180 μm acquire FSH-dependency. Androgen supports follicle development during the FSH-dependent preantral stage by enhancing FSH action via increased expression of *Fshr* mRNA levels. Androgen does not significantly affect the mRNA levels of several growth factors. However, simultaneous exposure of FSH and androgen causes preantral follicle degeneration due to another reason.
